# Radiological Insights of a Complex Orbital Blow-Out Fracture

**DOI:** 10.7759/cureus.74804

**Published:** 2024-11-30

**Authors:** Dania Ali, Omar Najim, Artur Wojciechowski

**Affiliations:** 1 Radiology, NHS, London, GBR; 2 Trauma and Orthopedics, Broomfield Hospital, London, GBR; 3 Radiology, NHS, Essex, GBR

**Keywords:** assault incident, blowout, facial trauma management, horizontal diplopia, orbital floor blowout fracture, orbital trauma surgery

## Abstract

CT is the gold standard for evaluating orbital trauma, providing rapid and detailed imaging of bony structures, soft tissue, and the globe. This is crucial in assessing orbital trauma due to its potential to cause significant impairment of ocular function. This case report presents a 35-year-old male who was admitted to the emergency department with a complicated left orbital blow-out fracture following blunt facial trauma. Initial assessment revealed symptoms of diplopia, periorbital discoloration, and orbital tenderness, which prompted further investigation through diagnostic imaging. CT imaging of the facial bones played a key role in confirming the diagnosis and detailing the extent of both bony injury and soft tissue involvement. These findings underscore the value of CT in evaluating the complexity of the injury, predicting potential complications, and guiding surgical and medical management by the care team.

## Introduction

Orbital fractures are common yet serious injuries in maxillofacial trauma, typically resulting from non-penetrating blunt force. The most common causes include road traffic accidents, followed by sports-related injuries and physical assaults, with the majority of cases presenting as uncomplicated fractures of the medial or floor orbital walls. If left untreated, these injuries can lead to both functional and cosmetic complications. There are two primary theories explaining the mechanisms behind these injuries: the buckling theory and the hydraulic theory. The buckling theory posits that force applied to the orbital rim is transmitted to weaker areas of the orbit, resulting in a fracture. In contrast, the hydraulic theory suggests that blunt facial trauma pushes the globe backward into the orbit, increasing pressure in the orbital cavity. This elevated pressure weakens the fragile bony walls, particularly the orbital floor and medial wall, leading to fracture [1].

Orbital trauma can cause significant and lasting damage. Minor fractures can entrap herniated soft tissues, leading to muscle and orbital fat ischemia, which manifests as pain, swelling, restricted ocular mobility, and delayed healing. Persistent ischemia may progress to tissue necrosis, resulting in permanent damage, disfigurement, or functional impairment. Larger fractures can displace the globe inferiorly and posteriorly, causing enophthalmos. Moreover, substantial soft tissue displacement into the fracture site may impair globe function, leading to hypoglobus, where globe supra-adduction and abduction are constrained, and causing symptoms like double vision [2].

In an emergency setting, clinical evaluation may be limited by soft tissue damage and the patient’s overall condition. In such cases, CT serves as the gold standard for assessing orbital fractures and associated soft tissue damage. It is also essential for guiding surgical planning and treatment management [3].

In this case, a 35-year-old male presented with a severe orbital wall fracture, which was unexpectedly complex given the level of assault he had endured. Despite the seemingly mild nature of the injury, the fracture involved significant damage to the orbital structure, primarily affecting the left orbit. He presented with a broad spectrum of symptoms affecting his left vision, indicating multilayered damage and hidden complexity. The heterogeneity of the injury warranted further evaluation with CT, which remains the ideal method for accurately diagnosing and assessing orbital trauma fractures [4].

## Case presentation

Initial presentation

A 35-year-old male presented to the emergency department after sustaining two blows to the face. He reported worsening pain in the left eye, accompanied by significant eyelid bruising and swelling. He also noted floaters, flashes, and blurring of vision, primarily in the left eye, with no visual symptoms in the right eye. In addition to the ocular symptoms, the patient described significant rhinalgia, nasal swelling, and a brief episode of epistaxis that resolved within ten minutes of injury. He denied experiencing any head injury, loss of consciousness, nausea, vomiting, weakness, or paraesthesia. His medical history was unremarkable, and he reported no history of smoking or alcohol consumption.

Physical examination

On examination, the patient was alert and oriented. Inspection of the left eye revealed significant periorbital ecchymosis, particularly at the medial canthus, accompanied by pronounced swelling and erythema. Notable mechanical ptosis of the left eye was observed, preventing voluntary elevation, and manual assistance was required to complete the examination. Palpation revealed multiple points of bony tenderness localized around the left orbital rim, including the supraorbital margin, infraorbital rim, medial canthal area, and lateral orbital wall. Assessment of extraocular muscle movement revealed no obvious limitations or pain on movement; however, vertical diplopia was noted exclusively in the left eye, exacerbated by upward gaze. The conjunctiva of the left eye was markedly injected, with a round 2 mm pupil that was reactive to light. The right eye was normal, with clear conjunctiva and a round, reactive pupil. No foreign objects were detected in either eye. Visual acuity was 6/6 in the right eye and 6/9 in the left eye using the near vision chart, indicating mild visual impairment in the left eye. There was no relative afferent pupillary defect. Examination of the nasal region revealed significant edema and tenderness over the nasal dorsum. No nasal hematoma or active epistaxis was noted.

CT imaging findings

**Figure 1:**
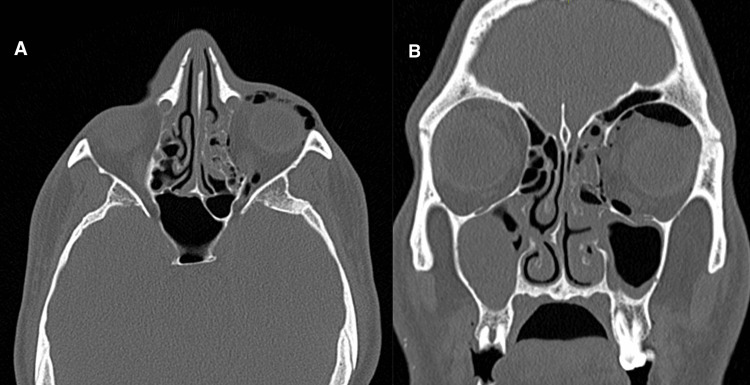
Coronal and axial views of the CT bone window Axial (A) and coronal (B) CT acquisitions with bone algorithms, demonstrating a blow-out fracture of the left orbital medial wall and left orbital floor.

The patient was stabilized, given adequate pain relief, and an urgent CT head and CT facial bones were arranged. The ophthalmological and maxillofacial teams were consulted due to concerns regarding significant orbital trauma. The CT head revealed no obvious intracranial pathology, with no evidence of skull base or calvarial fracture. However, the CT facial bones revealed a complex left orbital blow-out fracture with substantial medial wall deformity, as evidenced in Figure 1. The left blow-out fracture was characterized by the displacement of medial wall bony fragments toward the ethmoid cells, along with a displaced fracture of the lateral wall of the left orbit. Additionally, locules of air were visualized along the left orbital rim, extending anteriorly into the left upper and lower eyelids.

Management

An urgent same-day consultation was arranged with both the ophthalmology and maxillofacial teams, who conducted a comprehensive evaluation of the patient’s condition. They prescribed a course of topical antibiotics to mitigate the risk of infection resulting from ocular trauma. The maxillofacial team assessed the extent of the orbital injuries and contributed to the overall management plan. Surgical intervention was thoroughly discussed with the patient as a potential option; however, the patient opted against it. Follow-up arrangements were made, with an outpatient appointment scheduled in seven days to reassess the patient’s clinical progress and refine both medical and surgical plans as necessary.

## Discussion

Anatomy of the orbit

The pyramid-shaped orbit consists of four walls formed by seven distinct bones. The medial wall is made up of the lacrimal bone, ethmoid bone, maxilla, and lesser wing of the sphenoid, separating the orbital contents from the ethmoid sinuses. The lateral wall is composed of the greater wing of the sphenoid and the zygomatic bones. The floor, crucial for maintaining the orbit’s integrity, is formed by the maxilla and zygomatic bone, extending from the inferior orbital rim to the superior orbital fissure. The roof is primarily formed by the frontal bone, with part of the lesser wing of the sphenoid, separating the orbit from the frontal sinus and anterior cranial fossa.

The globe sits in the anterior portion of the orbit, with its distinct layers intertwined and indistinguishable on CT imaging in a healthy eye. The orbit is attached to seven extraocular muscles, which control eye movement, except for the levator palpebrae muscle, which elevates the upper eyelid. The optic nerve, a collection of axons from the retinal ganglion cells, travels through the center of the orbit and exits via the optic canal in the sphenoid bone. The ophthalmic artery, a branch of the internal carotid artery, provides the main blood supply to the orbit, while the superior ophthalmic vein drains it through the superior orbital fissure [5].

Orbital fracture types

Orbital fractures can involve one or more walls and may also occur as part of complex facial fracture patterns, such as Le Fort fractures and naso-orbito-ethmoid fractures. These fractures are commonly classified as blow-in, blow-up, or blow-out fractures. Blow-in fractures are characterized by upward displacement of the orbital floor, whereas blow-up fractures involve upward displacement of the orbital roof into the cranial cavity. Blow-out fractures, on the other hand, refer to the herniation of soft tissue out of the orbit [6].

Medial wall and orbital floor blow-out fractures

Orbital floor blow-out fractures are the most common type of orbital fractures, often occurring alone or in conjunction with other facial fractures. They typically result from direct blunt trauma to the globe and, in 50% of cases, are associated with medial wall fractures. The orbital walls vary in thickness, with the medial and orbital floors being thinner and more susceptible to injury. The medial wall is the thinnest, although the bony septa within the ethmoid sinus provide reinforcement and support to the fragile lamina papyracea, which slightly reduces the incidence of fractures in this area [7].

Orbital floor blow-out fractures are commonly characterized by the displacement of bone fragments into the maxillary sinus. A specific type of orbital floor fracture, known as a trapdoor fracture, occurs when orbital fat and/or the inferior rectus muscle herniate through the fracture into the maxillary sinus while the bone fragments fall back into place. This type of fracture is typically seen in children due to the malleability of their facial bones, which tend to spring back into position, creating a “trapdoor” effect [8].

**Figure 2:**
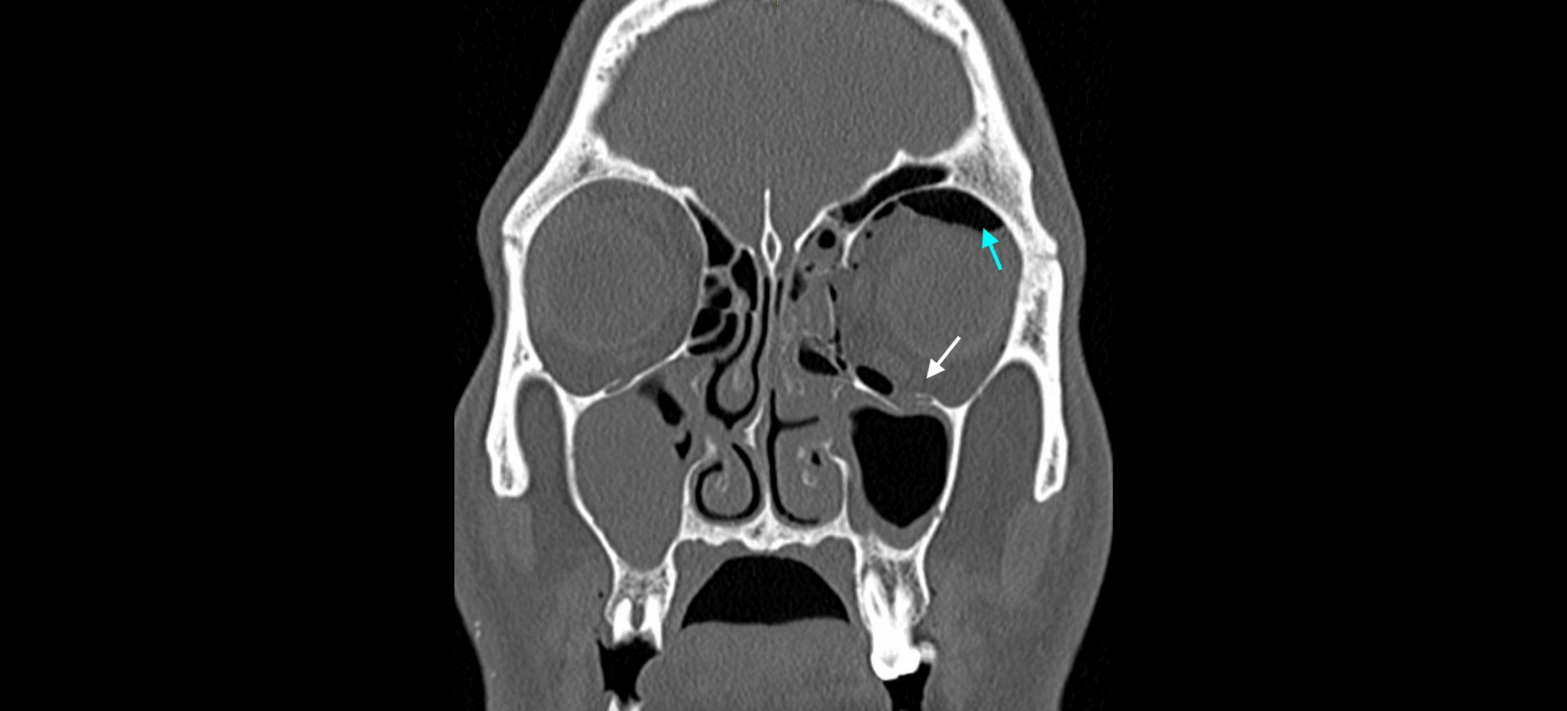
Coronal CT of the facial bones Coronal CT acquisition with bone algorithm showing a left orbital floor fracture with herniation of soft tissue into the maxillary sinus (white arrow) and the presence of intraorbital emphysema (blue arrow), a subtle sign of fracture.

In the case of this 35-year-old male, a left orbital fracture with displacement of the inferior rectus muscle into the maxillary sinus is visible in Figure 2. This complication is significant as it is a key factor in determining the need for surgical intervention. Furthermore, a study by Cellina et al. found that specific CT variables, such as extra-ocular muscle displacement and entrapment, strongly correlate with diplopia [9]. This aligns with our patient’s clinical presentation, who experienced vertical diplopia, particularly in upward gaze.

The second most common orbital fractures are those involving the medial wall, typically through the lamina papyracea. These fractures are often less obvious on imaging and may require attention to more subtle signs, such as orbital emphysema. On CT imaging, subtle medial wall fractures can be detected by comparing the distance from the midline to the medial wall on both the affected and unaffected sides. The fractured side will show a reduced distance compared to the unaffected side. Surgical intervention may be necessary in cases of ocular motility restriction due to muscle entrapment, diplopia, or significant enophthalmos. While enophthalmos is uncommon in isolated medial wall fractures, it may occur when associated with floor fractures [10].

**Figure 3:**
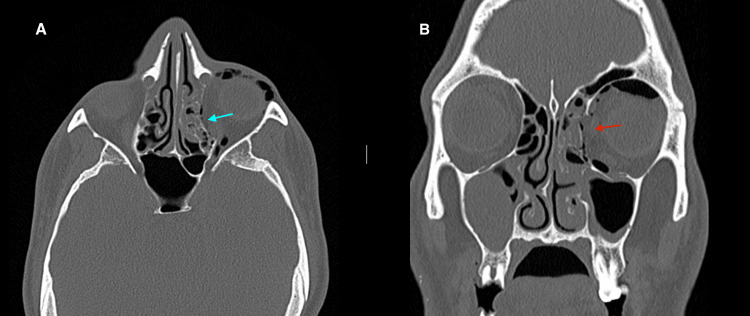
Axial and coronal CT of the facial bones Axial (A) and coronal (B) CT acquisitions with bone algorithms showing a large bone defect of the left lamina papyracea (red arrow) in the left medial wall fracture, with discontinuation of the lamina papyracea (blue arrow) and herniation of intraorbital fat tissue into the ethmoidal cells.

In Figure 3, there is clear distortion of the medial wall of the left orbit, with evidence of intraorbital emphysema (as shown in Figure 2), consistent with a medial wall fracture.

Further complications

Identifying foreign bodies within the globe is critical, as their retention increases the risk of infection in the internal structures of the eye. CT imaging is highly sensitive in detecting metallic foreign bodies at all depths within the globe and surrounding soft tissues, where they appear as hyper-densities with potential beam artifacts. However, CT is less sensitive to plastic foreign bodies and wooden objects. Wooden foreign bodies may appear hypoattenuating, resembling air, while plastics are often difficult to discern. In such cases, identifying signs of open globe injury is essential to guide diagnosis and management [11].

Open-globe injuries are severe and a leading cause of visual impairment. The imaging modality of choice for detecting these injuries remains CT, with a sensitivity greater than 70%. Key diagnostic features include a reduction in globe volume, the presence of intraorbital air, foreign bodies, and changes to the globe’s outline. Ruptures typically occur at the muscle insertions, where the sclera is most fragile, resulting in a globe that appears dysmorphic, with irregular borders and volume loss. It is important to note that open globe injuries can resemble other ocular conditions, such as colobomas or specific ophthalmological procedures, making accurate identification crucial for differentiation [12].

Corneal lacerations can be detected on CT by measuring the anterior-posterior diameter of the anterior chamber. A reduction in this diameter indicates decreased fluid volume, which is a key finding suggesting a potential penetrating corneal laceration. The anterior chamber may also develop a hyphema due to damage to blood vessels within the iris or ciliary body. This can be visualized on CT as an increased attenuation in the anterior portion of the globe, often correlating clinically. CT is also helpful in assessing the posterior chamber for hemorrhage, where clinical examination may not be possible. Vitreous hemorrhage appears as heterogeneous fluid density on CT, with areas of increased density indicating the presence of blood. Hyperdensity in the retrobulbar area may suggest retrobulbar hemorrhage, a crucial complication that can lead to orbital compartment syndrome if blood accumulates in the retrobulbar space [13].

Orbital compartment syndrome occurs when there is a sudden increase in pressure within the orbital space. Literature suggests that visual loss can occur after as little as 60 minutes of elevated orbital pressure. While orbital compartment syndrome is clinically diagnosed in acute settings, CT imaging is ideal for quick and accessible evaluation. CT can reveal tenting of the posterior globe, where the optic nerve insertion is located. The source of the syndrome, such as retrobulbar hemorrhage or space-occupying intraorbital foreign bodies, can often be identified. Recognizing any trauma to the optic nerve is vital for preserving vision. Although CT has limited value in diagnosing traumatic optic neuropathy, it can help localize fractures of the optic canal and detect optic nerve swelling. If the optic nerve is completely severed, aggressive management is usually not recommended [14].

Management of orbital blow-out fractures

The mid-face is a complex anatomical region, and unnecessary surgical interventions can lead to avoidable iatrogenic complications, such as trauma to the infraorbital nerve, postoperative diplopia, optic nerve damage, hemorrhages, and infections. Conversely, inadequate conservative management can result in long-term issues such as diplopia, enophthalmos, reduced ocular motility, and distressing aesthetic complications. Indications for orbital repair remain somewhat unclear but are typically guided by a combination of CT imaging findings and clinical assessment. The Burnstine criteria outline three major indications for immediate repair: (1) diplopia with CT evidence of entrapped muscle or periorbital tissue; (2) presence of a “white-eyed blow-out fracture” with CT evidence of compressed orbital tissue; and (3) early enophthalmos or hypoglobus with facial asymmetry [8].

A study by Schouman et al. in Geneva evaluated a CT-based method for predicting treatment strategies for orbital fractures. They identified the severity of inferior rectus muscle displacement seen on CT scans as a key independent predictor for surgical intervention [15]. In this case, the patient met the Burnstine criteria for surgery due to muscle entrapment, which caused diplopia. Surgical correction in such complex cases typically involves removing displaced bone fragments and reconstructing the orbital wall using a material with suitable biomechanical properties [16]. The goal is to release the entrapped muscle and restore the original orbital volume.

Despite being deemed to have capacity, the patient in this case declined surgery, citing social and practical concerns. Nevertheless, a structured follow-up plan was put in place to monitor her progress. This included regular ophthalmological evaluations to assess any changes in visual acuity, the development of enophthalmos, worsening diplopia, or functional impairment. Repeat imaging was scheduled to monitor the progression of soft tissue injury and to determine if further intervention would be necessary.

## Conclusions

This case highlights the key factors to consider when evaluating scans for patients presenting with orbital trauma. A thorough understanding of orbital anatomy, possible fracture types, and the structures most vulnerable to damage within the orbit is essential for accurate diagnosis and guiding effective treatment planning. The management of this patient involves careful long-term follow-up to monitor persistent symptoms, such as diplopia and painful eye movements, following the acute phase of the orbital blow-out fracture. If diplopia persists beyond one week, weekly follow-up appointments are scheduled. Once improvement plateaus, the option for surgical intervention is reintroduced. This structured approach ensures appropriate care and facilitates timely intervention when necessary.
